# Kappa opioid receptor activation alleviates experimental autoimmune encephalomyelitis and promotes oligodendrocyte-mediated remyelination

**DOI:** 10.1038/ncomms11120

**Published:** 2016-04-04

**Authors:** Changsheng Du, Yanhui Duan, Wei Wei, Yingying Cai, Hui Chai, Jie Lv, Xiling Du, Jian Zhu, Xin Xie

**Affiliations:** 1 Shanghai Key Laboratory of Signaling and Disease Research, Laboratory of Receptor-Based Bio-Medicine, School of Life Sciences and Technology, Tongji University, Shanghai 200092, China; 2 CAS Key Laboratory of Receptor Research, National Center for Drug Screening, Shanghai Institute of Materia Medica, Chinese Academy of Sciences, Shanghai 201203, China

## Abstract

Multiple sclerosis (MS) is characterized by autoimmune damage to the central nervous system. All the current drugs for MS target the immune system. Although effective in reducing new lesions, they have limited effects in preventing the progression of disability. Promoting oligodendrocyte-mediated remyelination and recovery of neurons are the new directions of MS therapy. The endogenous opioid system, consisting of MOR, DOR, KOR and their ligands, has been suggested to participate in the pathogenesis of MS. However, the exact receptor and mechanism remain elusive. Here we show that genetic deletion of KOR exacerbates experimental autoimmune encephalomyelitis, whereas activating KOR with agonists alleviates the symptoms. KOR does not affect immune cell differentiation and function. Instead, it promotes oligodendrocyte differentiation and myelination both *in vitro* and *in vivo*. Our study suggests that targeting KOR might be an intriguing way to develop new MS therapies that may complement the existing immunosuppressive approaches.

## Abstract (web summary)

Current treatments of multiple sclerosis are aimed at immunosuppression. Here the authors show that kappa opioid receptor is important for oligodendrocyte differentiation and myelination and the receptor agonists are protective in a mouse model of multiple sclerosis.

## Graphical abstract

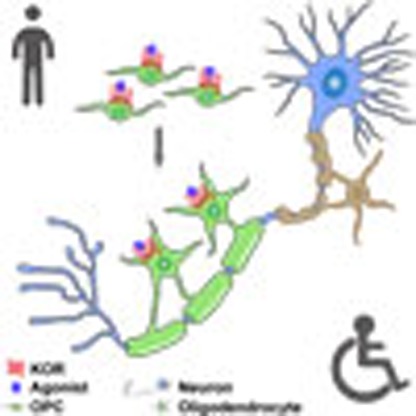

Multiple sclerosis (MS) is a predominantly T-cell-mediated autoimmune disorder that results in inflammatory damage to the central nervous system (CNS) and causes disability in young adults. The pathogenesis of MS is characterized by a cascade of pathological events, involving the activation of the immune system, infiltration of lymphocytes, activation of microglia, focal inflammatory demyelination and axonal damage^1^. CD4^+^ T cells, especially the Th17 and Th1 subgroups, have been suggested to cause the early initiation of the disease^2^.

All the currently available treatments for MS target the immune system with mechanisms of action including general immunosuppression/immunomodulation, such as beta-interferons, glatiramer acetate, mitoxantrone, teriflunomide, fingolimod and dimethyl fumerate, and blockade of immune cell infiltration into the CNS, such as natalizumab^3^. Although effective in reducing the relapse rate and the formation of new lesions, these drugs, however, have very limited effects in preventing the progression of disability. Promoting oligodendrocyte progenitor cell (OPC) differentiation, remyelination and subsequent functional recovery of the neurons have been proposed to be the new direction of MS therapy^4^,^5^.

The endogenous opioid system has been suggested to play a role in the pathogenesis of MS. In a Theiler's murine encephalomyelitis virus model of MS, mRNA levels of all the three opioid receptors, that is, the mu, delta and kappa opioid receptors (MOR, DOR and KOR), were significantly decreased in the spinal cord^6^. The loss of opioid receptors might partially explain the central neuropathic pain commonly observed in the MS patients^7^. Pregnant women have been reported to express higher levels of endogenous opioids^8^. Pregnant MS patients experience remission of the disease and have fewer relapses. However, 3 months after delivery, these women show a marked increase in relapse rate, in contrast to the decrease in endogenous opioid levels^9^,^10^. A pilot clinical study showed that met-enkephalin given intrathecally exerted beneficial effects on 13 patients with chronic severe progressive MS^11^, indicating that activating the opioid receptors might be beneficial. However, several small-scale clinical studies showed that low-dose naltrexone (LDN), a non-selective opioid receptor antagonist, also had protective effects on MS patients^12^,^13^,^14^. Although the exact mechanism remains unclear, LDN may block opioid receptors intermittently and thus promote the expression of opioid peptides and receptors^15^.

The endogenous opioid responses are mainly mediated by MOR, DOR and KOR, all of which are G-protein-coupled receptors. The previous studies indicate the involvement of opioid system in MS without knowing the exact receptor and mechanism. Here we study the experimental autoimmune encephalomyelitis (EAE), a commonly used animal model of MS, in MOR, DOR or KOR knockout mice, and find genetic deletion of KOR induces a significantly severer phenotype of EAE. KOR does not affect T-cell differentiation and function. Instead, it is critically involved in the differentiation of OPC towards myelinating oligodendrocytes (OLs). Activating KOR promotes OPC differentiation and remyelination, whereas KOR knockout prevents agonist-mediated beneficial effects.

## Results

### KOR knockout mice are more susceptible to EAE

We first examined the mRNA levels of the three opioid receptors (DOR, KOR and MOR) and the precursors of endogenous opioids including proenkephalin (Penk) and prodynorphin (Pdyn) in MOG-EAE mice on various days post immunization. In the brain, the expression levels of these genes did not show a clear time-dependent change (Supplementary Fig. 1a). In the spinal cord, however, all the genes were significantly downregulated from day 15, which is typically after the onset of the disease (Supplementary Fig. 1b), similar as previously reported in a Theiler's murine encephalomyelitis virus model of MS^6^. In the lymph node, DOR was downregulated from day 9 to 18, while KOR did not show a time-dependent change. Owing to the low expression level, MOR, Penk and Pdyn were undetectable in the lymph node (Supplementary Fig. 1c). The downregulation of the receptors and ligands after MOG_35–55_ immunization indicates that the opioid receptors might be involved in EAE pathogenesis.

**Figure 1:**
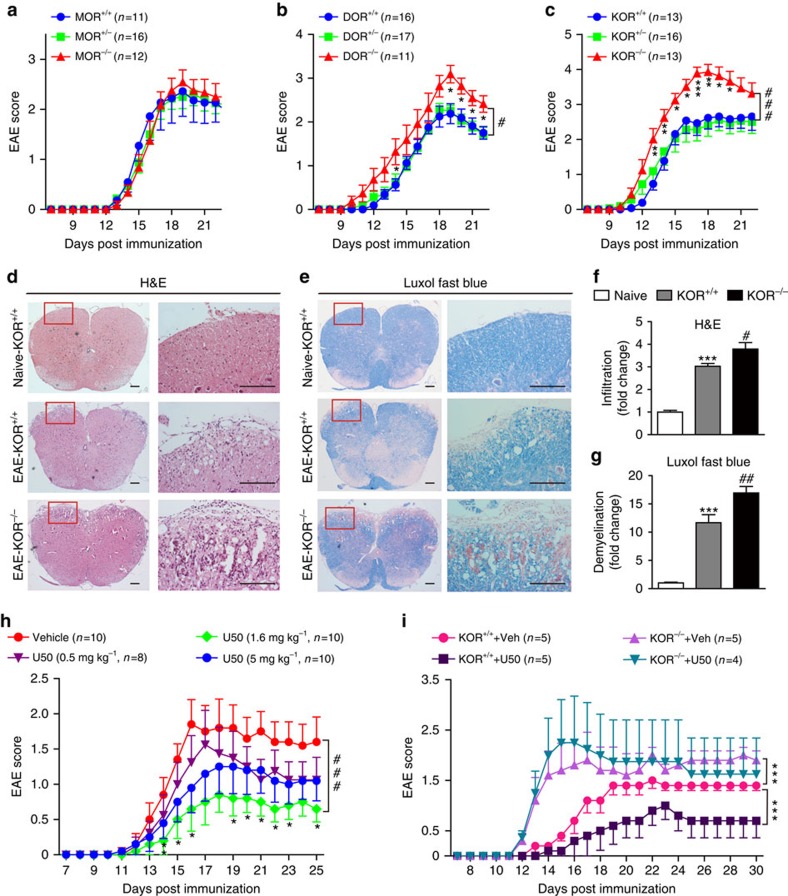
Opioid receptors are involved in the pathogenesis of EAE. (**a**–**c**) Clinical scores of EAE induced in WT, MOR^+/−^, MOR^−/−^ (**a**), DOR^+/−^, DOR^−/−^ (**b**), KOR^+/−^, KOR^−/−^ (**c**). Data are means±s.e.m. ^#^*P<*0.05, ^###^*P<*0.001 (two-way analysis of variance (ANOVA) test), *P<0.05, **P<0.01 and ***P<0.001 versus WT (Mann–Whitney *U*-test). (**d**) Haematoxylin and eosin and (**e**) Luxol fast blue staining of the paraffin sections of the spinal cords isolated from naive, WT-EAE or KOR^−/−^-EAE mice on day 17 post immunization. Scale bars, 200 μm. (**f**,**g**) Quantification of CNS infiltrates and the amount of demyelination presented in **d**,**e**. Three mice from each group were killed, and 15 sections from each mouse were analysed. Data are means±s.e.m. ***P<0.001 versus naive, ^#^*P<*0.05, ^##^*P<*0.01 versus WT-EAE (Student's *t*-test). (**h**) Clinical scores of WT-EAE mice treated with KOR agonist U50488 (0.5, 1.6 or 5 mg kg^−1^) or vehicle once daily via intraperitoneal injection from day 3 post immunization till the end of the study. ^###^*P<*0.001 (two-way ANOVA test), *P<0.05, **P<0.01 versus WT (Mann–Whitney *U*-test). (**i**) Clinical scores of WT- or KOR^−/−^-EAE mice treated with U50488 (1.6 mg kg^−1^) or vehicle once daily via intraperitoneal from day 3 post immunization. Data are means±s.e.m. ***P<0.001 (two-way ANOVA test).

We then induced EAE in wild-type (WT), opioid receptors knockout and heterozygous mice. Deletion of MOR did not seem to affect the onset of EAE (Fig. 1a). However, the deletion of DOR led to a slightly severer type of EAE (Fig. 1b), whereas ablation of KOR induced a significant increase in the disease score (Fig. 1c). The disease severity in heterozygotes was similar to their WT littermates (Fig. 1b,c). Histological examination of the spinal cords was performed on day 17 post immunization. Leukocytes infiltration and demyelination could be observed in the spinal cord of the WT (KOR^+/+^)-EAE animals (Fig. 1d–g). KOR^−/−^-EAE mice exhibited significantly increased leukocytes infiltration and more extensive demyelination (Fig. 1d–g).

### Pharmacological activation of KOR alleviates EAE

Since the knockout of KOR led to a severer type of EAE, we wondered whether activation of KOR confers protection against EAE. U50488 and asimadoline, two selective KOR agonists, were tested. U50488 had significant therapeutic effects at all the three dosages tested, with the best effect observed in the 1.6 mg kg^−1^ group, which showed the lowest peak severity and cumulative clinical score (Fig. 1h). Asimadoline also showed significant therapeutic effect (Supplementary Fig. 2). Histological analysis revealed that U50488 (1.6 mg kg^−1^) also significantly reduced leukocytes infiltration into the spinal cord and demyelination in EAE (Supplementary Fig. 3). To avoid the possible off-target effect of the agonist, U50488 (1.6 mg kg^−1^) was used to treat EAE induced in both the WT and KOR^−/−^ mice. Similar to our previous observations, U50488 significantly reduced EAE severity in the WT mice, and KOR knockout significantly increased the disease score. However, the therapeutic effect of U50488 was completely abolished in the KOR^−/−^-EAE mice (Fig. 1i). These data demonstrate that KOR signalling contributes to EAE resistance.

### KOR regulates EAE pathogenesis in CNS

**Figure 2:**
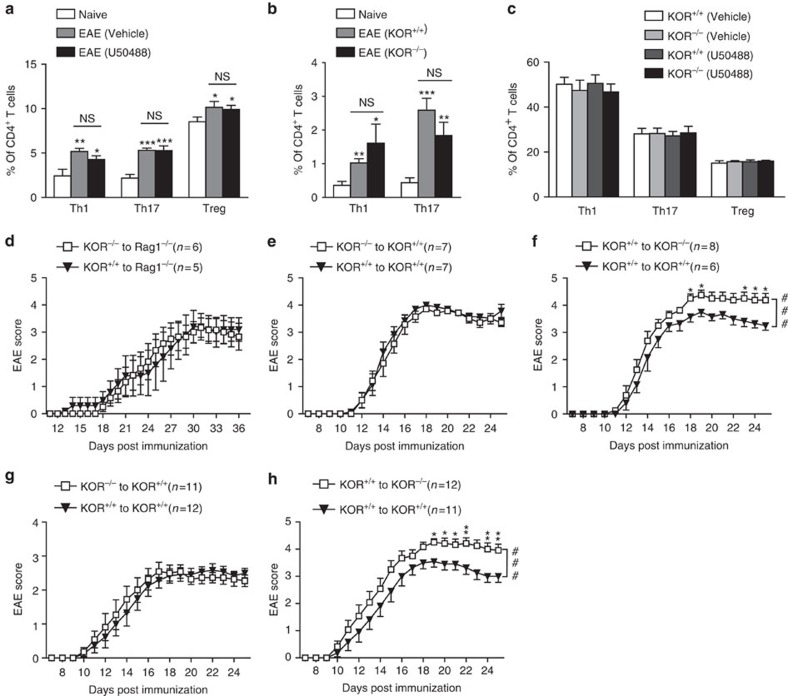
KOR in immune cells does not contribute to EAE pathogenesis. (**a**) Analysis of CD4^+^ T cell subtypes in the spleen of naive, WT-EAE and WT-EAE mice treated with U50488 (1.6 mg kg^−1^) on day 12 post immunization. Th1, Th17 and Treg cells were analysed with FACS by intracellular staining of IFN-γ, IL-17A and Foxp3, respectively. (**b**) Analysis of Th1 and Th17 cells in the spleen of naive, WT-EAE or KOR^−/−^-EAE mice on day 12 post immunization. (**c**) *In vitro* differentiation of Th1, Th17 and Treg cells from WT or KOR^−/−^ CD4^+^ T cells in the presence of U50488 (10 μM) or not. Data are means±s.e.m. (*n*=3), *P<0.05, **P<0.01, ***P<0.001 versus naive mice (Student's *t*-test). (**d**) EAE scores of Rag1^−/−^ mice reconstituted with splenocytes (1 × 10^7^) from WT or KOR^−/−^ mice and immunized with MOG_35–55_. (**e**) EAE scores of lethally irradiated WT mice reconstituted with WT or KOR^−/−^ bone marrow cells (1 × 10^7^) and immunized with MOG_35–55_. (**f**) EAE scores of lethally irradiated WT or KOR^−/−^ mice reconstituted with WT bone marrow cells and immunized with MOG_35–55_. ^###^*P<*0.001 (two-way analysis of variance (ANOVA) test), *P<0.05, KOR^−/−^ versus KOR^+/+^ recipients (Mann–Whitney *U*-test). (**g**) Clinical scores of passive EAE in WT mice induced by transferring MOG-restimulated splenocytes (1 × 10^7^) isolated from WT or KOR^−/−^ EAE mice. (**h**) Clinical scores of passive EAE in WT or KOR^−/−^ mice induced by transferring of MOG-restimulated splenocytes (1 × 10^7^) isolated from WT EAE mice. ^###^*P<*0.001 (two-way ANOVA test), *P<0.05, **P<0.05, KOR^−/−^ versus KOR^+/+^ recipients (Mann–Whitney *U*-test). Data are means±s.e.m.

MS is an autoimmune disease initiated by immune cells in the periphery. T cells, B cells and monocytes have all been reported to participate in MS pathogenesis^16^,^17^,^18^. However, U50488 did not alter the percentage of CD4^+^ T cells, CD8^+^ T cells, B cells and CD11b^+^ cells in the spleen, blood and lymph node of EAE mice (Supplementary Fig. 4a). Similarly, KOR knockout did not significantly affect the percentage of these cells except that the CD8^+^ T cells were slightly reduced (Supplementary Fig. 4b). The percentage of the major pathogenic Th1 and Th17 subgroups of the CD4^+^ T cells in EAE mice were also not affected by the treatment of U50488 (Fig. 2a and Supplementary Fig. 5a) or KOR knockout (Fig. 2b and Supplementary Fig. 5b). Cytokine secretion by Th1 and Th17 cells (IFN-γ and IL-17A) after MOG_35–55_ restimulation was not affected by U50488 or KOR knockout either (Supplementary Fig. 5c,d). *In vitro* differentiation of Th1, Th17 and Treg cells were also not affected by KOR activation or knockout (Fig. 2c).

These observations led us to speculate that KOR might not function in the immune system during EAE pathogenesis. We induced EAE in Rag1^−/−^ mice reconstituted with splenocytes isolated from WT or KOR^−/−^ animals. KOR deficiency in the splenocytes did not affect the EAE score in these Rag1^−/−^ mice (Fig. 2d). Bone marrow chimeras were generated by transferring WT or KOR^−/−^ bone marrow cells to lethally irradiated WT mice, or transferring WT cells to lethally irradiated WT or KOR^−/−^mice (Fig. 2e,f). EAE were induced after immune system reconstitution. KOR deletion in the bone marrow cells did not affect the EAE score in these chimeras (Fig. 2e). However, a significant increase in the disease score was observed when KOR was knocked out in the recipient mice (Fig. 2f). Passive EAE was also induced by adoptive transfer of splenocytes isolated from MOG_35–55_ immunized WT or KOR^−/−^ mice to WT mice, or from MOG_35–55_ immunized WT mice to WT or KOR^−/−^ mice (Fig. 2g,h). KOR deletion in the splenocytes did not affect the passive induction of EAE (Fig. 2g), while KOR deletion in the recipient mice significantly enhanced the disease severity (Fig. 2h). These data demonstrate that KOR in the immune system is not involved in EAE pathogenesis, while KOR in the CNS might play a critical role in controlling the disease severity.

### KOR does not block astrocytes and microglia activation

It has been reported that the brain endothelial cells, astrocytes and microglia participate in the leukocyte trans-migration and inflammatory process in the CNS, leading to the onset of EAE^19^,^20^,^21^. So we explored whether KOR signalling affects the activation of these cells. The mouse brain microvascular endothelial bEnd.3 cells could be activated by TNF-α and INF-γ stimulation^22^,^23^, and could upregulate the expression of cell adhesion molecules and chemokines, including ICAM1, VCAM1, CCL2, CCL5 and IP-10, and downregulate the tight junction protein Occludin (Supplementary Fig. 6). These changes may facilitate the breakdown of blood–brain barrier and the infiltration of leukocytes. KOR agonist U50488 could not block TNF-α- and INF-γ-induced downregulation of Occludin, it even enhanced cell adhesion molecule and chemokine expression, indicating that the beneficial effect of KOR activation in EAE may not relate to endothelial cell regulation. The primary astrocytes were isolated and activated *in vitro* by various cytokine combinations including TNF-α/IL17 and TNF-α/INF-γ (ref. 24). U50488 treatment did not seem to affect the expression of inflammatory cytokines and chemokines in activated astrocytes (Supplementary Fig. 7). Primary microglia were also isolated and activated *in vitro* by LPS^25^. U50488 even slightly enhanced LPS-induced inflammatory cytokine expression in microglia (Supplementary Fig. 8). Taken together, these data indicate that KOR signalling does not alleviate EAE by blocking the activation of brain vascular endothelial cells, microglia and astrocytes.

### KOR activation promotes remyelination in mouse models

**Figure 3:**
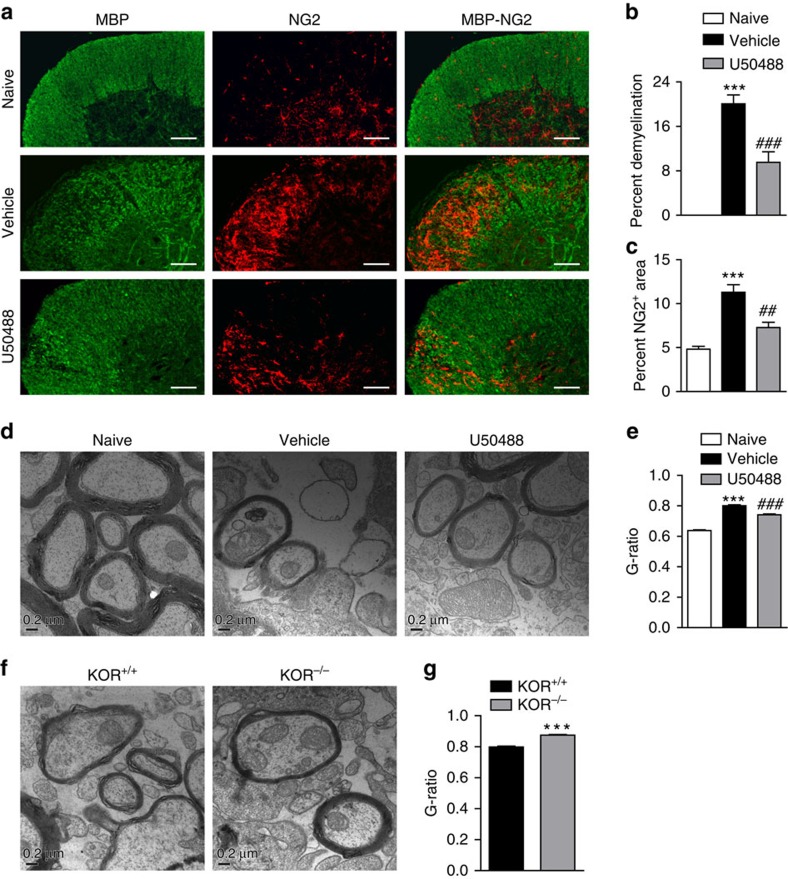
Activation of KOR promotes remyelination in EAE mice. (**a**) Immunofluorescence staining of OLs (MBP) and OPC (NG2) in the spinal cords isolated on day 22 post immunization from WT-EAE mice treated with vehicle or U50488 (1.6 mg kg^−1^). Scale bars, 100 μm. (**b**,**c**) Statistical analysis of the demyelination area and NG2 positive area in white matter. Three animals from each group were killed and 15 sections of the spinal cord of each animal were analysed. Data are means±s.e.m. ***P<0.001 versus naive group, ^##^*P<*0.01, ^###^*P<*0.001 versus vehicle group (Student's *t*-test). (**d**) Representative electron microscopy images of cross sections of the spinal cords isolated from naive, vehicle or U50488 (1.6 mg kg^−1^)-treated EAE mice on day 22 post immunization. Scale bar, 0.2 μm. (**e**) G-ratios of spinal cord axons in **d**. Data are means±s.e.m. (*n*=200), ***P<0.001 versus naive, ^###^*P<*0.001 versus vehicle treatment (Student's *t*-test). (**f**) Representative electron microscopy images of spinal cords isolated from WT or KOR^−/−^ EAE mice on day 22 post immunization. Scale bar, 0.2 μm. (**g**) G-ratios of spinal cord axons in **f**. Data are means±s.e.m. (*n*=200), ***P<0.001 versus KOR^+/+^ (Student's *t*-test).

MS and EAE are characterized by autoimmune-mediated demyelination and neurodegeneration. In CNS, myelin is formed by OLs. Current research on MS therapy is directed at three major goals: controlling the inflammatory immune response to prevent the development of new demyelinating lesions, protecting the demyelinated neurons from degeneration, and promoting OL differentiation and remyelination^26^. Remyelination can occur effectively and spontaneously following demyelination with the migration of OPCs to the sites of injury and subsequent differentiation to mature OLs that remyelinate the damaged axons. However, for reasons yet to be fully elucidated, this process is generally incomplete and often fails in MS^27^. Since KOR did not seem to play a role in the immune system, we wondered whether it could affect the remyelination process. The OLs and OPCs in the EAE lesions in the spinal cord were assessed by immunostaining using antibodies target MBP (OLs) or NG2 (OPCs) (Fig. 3a). MBP staining was substantially reduced in the EAE animals, indicating severe demyelination. The demyelinating areas were filled with NG2^+^ OPCs, indicating that the recruitment of OPCs to the lesions was normal but differentiation to OLs might be blocked (Fig. 3a–c). In U50488-treated EAE mice, demyelination was significantly mitigated, and NG2^+^ OPCs were also reduced (Fig. 3a–c). Furthermore, myelin surrounding spinal cord axons in the remission phase of EAE was observed with electron microscopy. Demyelination was very apparent and g-ratio of the myelinated axons was significantly increased in vehicle-treated EAE mice compared with the naive ones, but g-ratio in U50488-treated mice were significantly lower than that in vehicle-treated mice (Fig. 3d,e), indicating a possible better recovery. In contrast, the g-ratio in KOR^−/−^-EAE mice was significantly higher than WT-EAE animals, indicating less recovery (Fig. 3f,g). These observations suggested that KOR activation might promote the generation of OLs and remyelination in EAE.

**Figure 4:**
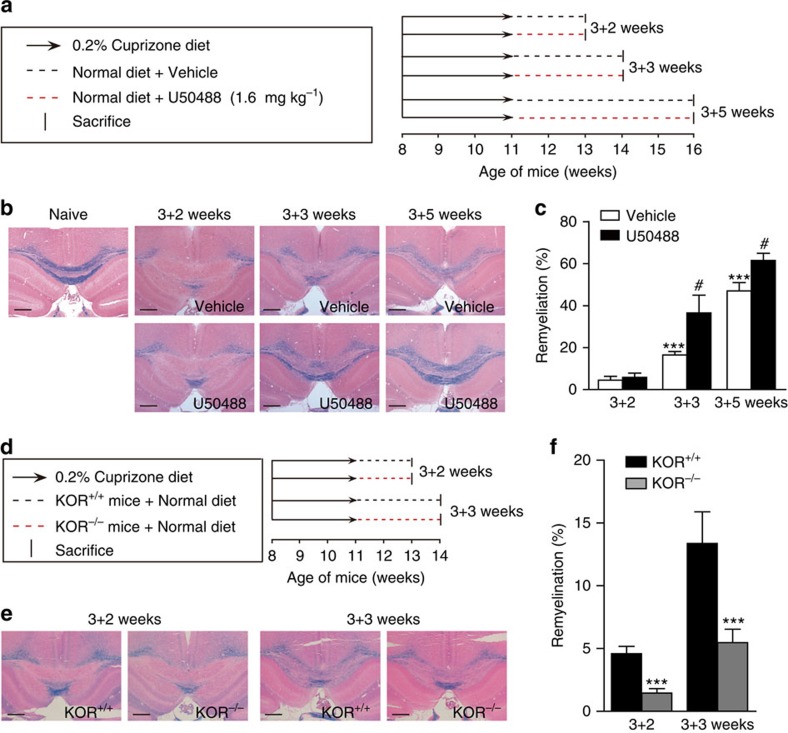
Activation of KOR promotes remyelination in cuprizone-induced demyelination mice model. (**a**) Demyelination was induced in WT C57BL/6 mice by feeding with a 0.2% cuprizone containing diet for 3 weeks. Following cuprizone withdrawal, the mice were treated with vehicle or U50488 (1.6 mg kg^−1^) for 2, 3 or 5 weeks (wks). (**b**) Representative images of the corpus callosum region stained with Luxol fast blue after cuprizone and U50488 treatment. Scale bars, 500 μm. (**c**) Statistical analysis of the myelinating areas in **b**. Six animals from each group were killed and six sections of the corpus callosum region of each animal were analysed. ***P<0.001 versus 2 wks treatment group, ^#^*P<*0.05 versus vehicle group (Student's *t*-test). (**d**) Demyelination was induced in KOR^+/+^ or KOR^−/−^ C57BL/6 mice by feeding with a 0.2% cuprizone containing diet for 3 wks. Following cuprizone withdrawal, the KOR^+/+^ or KOR^−/−^ mice were fed with a normal diet for 2 or 3 wks. (**e**) Representative images of the corpus callosum region from KOR^+/+^ or KOR^−/−^ mice stained with Luxol fast blue after the cuprizone treatment. Scale bars, 500 μm. (**f**) Statistical analysis of the myelinating areas in **e**. Six animals from each group were killed and six sections of the corpus callosum region of each animal were analysed. ***P<0.001 versus KOR^+/+^ (Student's *t*-test).

KOR-promoted remyelination was further evaluated with a non-immune-mediated, cuprizone-induced demyelination model^28^ (Fig. 4a). Myelin status at the corpus callosum was evaluated with Luxol fast blue staining. All cuprizone-fed groups showed a significant loss of myelin at the corpus callosum region (Fig. 4b). Two weeks after cuprizone withdrawal, both the vehicle and U50488 groups showed limited remyelination (Fig. 4b,c). However, significant spontaneous remyelination could be observed at 3 or 5 weeks after cuprizone withdrawal, and U50488 treatment further enhanced the remyelination process (Fig. 4b,c). We also checked the effect of KOR knockout in the cuprizone model, and the KOR^−/−^ mice showed significantly less remyelination than the WT mice (Fig. 4d–f).

### KOR promotes OL differentiation and myelination *in vitro*

**Figure 5:**
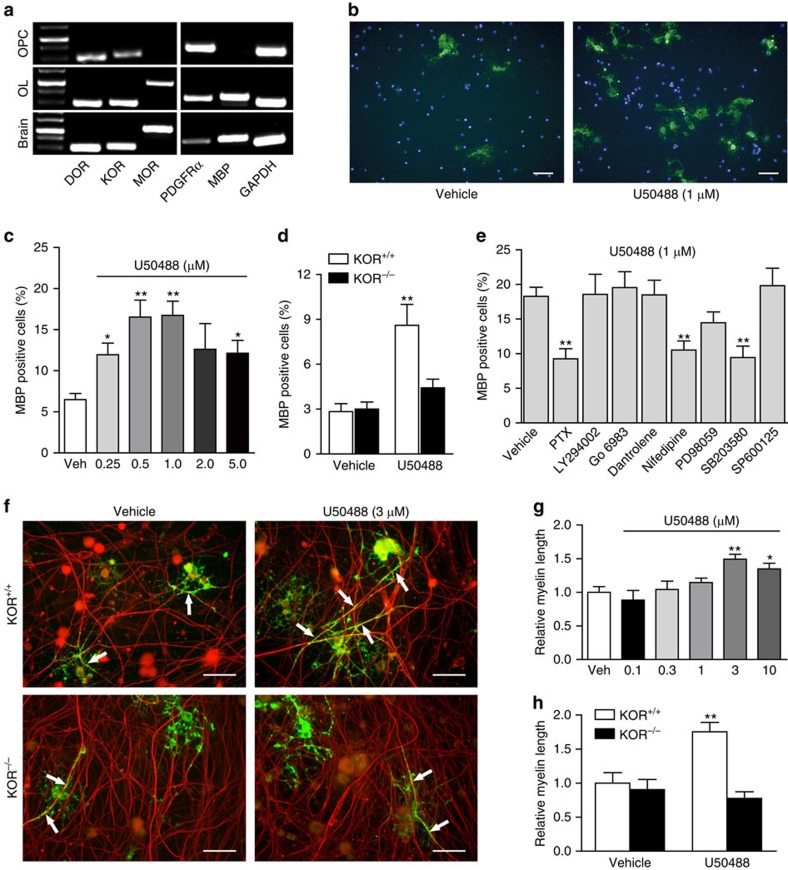
Activation of KOR stimulates oligodendrocyte differentiation and myelination *in vitro.* (**a**) PCR analysis of opioid receptors and cell-specific markers in OPC and OLs. Scale bars, 50 μm. (**b**,**c**) *In vitro* differentiation of OLs from OPCs in the presence of various concentrations of U50488. OLs were stained with antibody against MBP (**b**), and the dose-response of U50488 was presented in **c**. (**d**) OL differentiation in WT or KOR^−/−^ OPCs in the presence or absence of U50488. (**e**) The effects of various pathway inhibitors in U50488-stimulated OPC to OL differentiation. (**f**) OPCs isolated from KOR^+/+^ or KOR^−/−^ mice were co-cultured with DRG-neurons isolated from KOR^+/+^ mice and treated with vehicle or U50488. The cells were then immunostained for NF-200 (neurofilament, red) and MBP (oligodendrocytes, green). Arrows indicate myelinated axons (double positive for NF-200 and MBP). Scale bars, 50 μm. (**g**) Statistical analysis of myelinated axons in co-cultures containing KOR^+/+^ OPCs in the presence of various concentrations of U50488. (**h**) Statistical analysis of myelinated axons in co-cultures containing KOR^+/+^ or KOR^−/−^ OPCs in the presence or absence of U50488 (3 μM). Data are means±s.e.m., *P<0.05, **P<0.01 versus vehicle (Student's *t*-test).

To test whether this remyelination promoting effect of KOR is direct or not, and to rule out the possible crosstalk between neurons or other types of the cells in CNS with OPCs or OLs, we performed the *in vitro* OPC to OL differentiation assay. KOR was indeed expressed by the OPCs (Fig. 5a). And U50488 dose dependently promoted OL differentiation from OPCs, with the best effect appearing at 0.5 and 1 μM (Fig. 5b,c). This KOR agonist-mediated enhancement of OL differentiation was almost completely abolished in the KOR knockout OPCs (Fig. 5d). The downstream pathways linking KOR activation to OL differentiation were further analysed with pathway inhibitors. Blocking Gαi/o pathway with pertussis toxin (PTX), L-type calcium channels with nifedipine, or p38 pathway with SB203580 all significantly reduced U50488-promoted OL differentiation, while other pathway inhibitors showed no significant effect (Fig. 5e). The observation that KOR signalling promotes OPC differentiation towards OL in culture raises the question of whether it also promotes myelin formation. To address this question, we set up an *in vitro* myelination system^29^ by co-culturing OPCs isolated from KOR^+/+^ or KOR^−/−^ mice with dorsal root ganglion (DRG) neurons isolated from KOR^+/+^ mice. Myelinated axons will be positive for both the OL marker MBP and axon marker NF-200 (neurofilament, 200 kD). Compared with the control, administration of KOR agonist U50488 significantly increased the length of myelinated axons in the co-culture containing the KOR^+/+^ OPCs (Fig. 5f,g). And the positive effect of U50488 was abolished in the co-culture containing the KOR^−/−^ OPCs (Fig. 5f,h). Taken together, these results suggest that KOR activation enhances OL differentiation and myelination both *in vitro* and *in vivo.*

## Discussion

It has been known for a long time that opioids can influence the immune responses, typically exibiting inhibitory effects on humoral and cellular immune responses^30^. Early studies suggest that opioids might modulate immune responses indirectly via the hypothalamic–pituitary–adrenal axis^31^. But with the discovery of opioid receptors in various types of immune cells, direct modulation of immune cell functions by opioids have also been reported^32^. The role of opioid receptors in EAE was elusive. As a matter of fact, both agonists and low-dose antagonist (naltrexone, LDN) were found to be effective in treating MS or EAE^33^. The therapeutic effect of LDN was presumably explained by the feedback upregulation of endogenous opioids and opioid receptors caused by the temporary blockade of the receptors, but the exact mechanisms and receptors involved remain unclear. In this study, with knockout mice, we clearly demonstrate that MOR does not affect EAE, while knockout of DOR slightly enhances EAE, and genetic deletion of KOR induces a significantly severer type of EAE. And interestingly, KOR does not seem to modulate the disease via the immune system.

Apart from immune suppression, remission in MS/EAE also attributes to the migration of OPCs to the sites of demyelination and subsequent differentiation to mature oligodendrocytes which remyelinate the axon^5^,^34^. Our study indicates that activation of KOR stimulates OPC differentiation both *in vitro* and *in vivo*, and promotes remyelination in both immune- and non-immune-mediated demyelination mouse models. Although our data suggest that KOR does not directly affect the functions of major immune cell types during EAE induction, KOR-mediated remyelination and tissue repair might reduce secondary inflammation in CNS (Fig. 1d,f). A few earlier studies suggested that KOR is involved in the regulation of cytokine production in macrophage^35^,^36^. However, CD4^+^ T cells, and to a lesser extent, CD8^+^ T cells and B cells, are considered the major pathogenic cells in MS/EAE^2^,^37^. It is not surprising that the regulatory effect of KOR on macrophage might not significantly affect EAE pathogenesis.

Promoting OPC differentiation, remyelination and functional recovery of the neurons have recently been proposed to be the new direction of MS therapy^4^,^5^, since the immune suppressive drugs are not very effective in preventing the progression of disability. In a recent study, by using an image-based screen, Deshmukh *et al*.^34^ identified that benztropine, by blocking M1 and/or M3 muscarinic receptors, selectively induces OPC differentiation towards oligodendrocytes and promotes remyelination in EAE and cuprizone-induced demyelination model.

In conclusion, we demonstrate for the first time, to our knowledge, that the activation of KOR significantly alleviates the symptoms of EAE by promoting OPC differentiation and remyelination. Our study suggests that targeting KOR might be an intriguing way to develop effective new therapies for the treatment of MS that complement the existing immunosuppressive approaches.

## Methods

### Mice

C57BL/6 mice were purchased from Shanghai Laboratory Animal Center (Shanghai, China), DOR^−/−^, KOR^−/−^, MOR^−/−^ and Rag1^−/−^ mice in C57BL/6J background were all purchased from the Jackson Laboratory. All the mice were maintained in pathogen-free conditions with standard laboratory chow and water *ad libitum* and genotyped by PCR of DNA isolated from tail clippings. All the experiments were approved and conducted in accordance with the guidelines of the Animal Care Committee of Tongji University.

### Induction and assessment of EAE

Mice (8–10 weeks) were immunized subcutaneously with 200 μg MOG_35–55_ emulsified in CFA, which contains 5 mg ml^−1^ Mycobacterium tuberculosis. The mice received intraperitoneal injections with 200 ng PTX on days 0 and 2. For drug treatment, the mice receive daily intraperitoneal injection of U50488 or asimadoline starting from day 3. The disease severity was scored daily.

For adoptive transfer EAE, donor mice were immunized as described above. Splenocytes were prepared from donor mice on day 10 post immunization and restimulated *in vitro* with MOG peptide at a concentration of 25 μg ml^−1^ for 72 h. A total of 1 × 10^7^ splenocytes per mouse was transferred intravenously into recipients that were sublethally irradiated (4 Gy) before adoptive transfer (day 0). On days 0 and 2, the animals were also injected intraperitoneally with 200 ng PTX.

The mice were then killed at the end of EAE clinical score assessment or at the time point as indicated in the text.

### Chimera generation and EAE induction

In some experiments, Rag1^−/−^ mice received 1 × 10^7^ splenocytes from WT or KOR^−/−^ mice from the same litter 1 day before immunization. The recipient mice were immunized for the induction of active EAE, as described above. For bone marrow chimeras, 5-to-6-week-old female WT or KOR^−/−^ mice were irradiated with a lethal dose of 8 Gy, the bone marrow cells from 6-week-old WT or KOR^−/−^ mice from the same litter were then transferred intravenously into the irradiated mice (1 × 10^7^ cells per mouse) on the same day. Six weeks later, these bone marrow chimeras were subjected to EAE induction with MOG immunization.

### Cuprizone-induced demyelination mouse model

Female C57BL/6 mice (8 weeks) were fed with 0.2% (w:w) cuprizone (Sigma) mixed into ground standard rodent chow. Cuprizone diet was maintained for 3 weeks, thereafter cuprizone-infused feed was removed and the animals were given standard chow. The mice receive daily intraperitoneal injection of U50488 (1.6 mg kg^−1^) after cuprizone withdrawal. The treatment lasted for 2, 3 or 5 weeks. The animals were then killed. Their brains were extracted, paraffin-embedded, sectioned and stained for histopathological analysis.

### Histopathological and immunohistochemical analysis

Paraffin-embedded sections of the spinal cords and brains were stained with haematoxylin and eosin or Luxol fast blue to visualize leukocyte infiltration or assess demyelination, respectively. For immunohistochemistry, frozen sections of the spinal cords were incubated with Rabbit polyclonal anti-NG2 antibody (Millipore, AB5320, 1:200) and mouse anti-MBP antibody (Covance, SMI-94R, 1:500) at 4 °C overnight. After thorough washing, the sections were stained with secondary antibody conjugated to Alexa Fluor 488 (Thermo Fisher, A-11001, 1:1,000) or 546 (Thermo Fisher, A-11010, 1:1,000), and nuclei were stained with Hoechst 33342. Images were taken using an Olympus IX51 inverted fluorescent microscope or an Olympus FV10i confocal microscope, and quantitative image analysis was performed using ImagePro.

### Flow cytometry

Lymph node cells, peripheral blood monocytes and splenocytes were stimulated with PMA (50 ng ml^−1^), ionomycine (750 ng ml^−1^) and brefeldin A (10 μg ml^−1^) for 5 h at 37 °C. Surface markers (CD4, CD8, B220 and CD11b) were stained with relevant antibodies (CD4 (BioLegend, 100528, 1:100), CD8 (eBioscience, 11-0081, 1:100), B220 (eBioscience, 12-0452, 1:100) and CD11b (eBioscience, 11-0112, 1:100)). Following surface staining, the cells were resuspended in fixation/permeabilization solution (Cytofix/Cytoperm kit; BD Bioscience) and intracellular IL-17A and IFN-γ were stained with PE-labelled anti-mouse IL-17A antibody (BioLegend, 506904, 1:100) and APC-labelled anti-mouse IFN-γ antibody (BioLegend, 505810, 1:100), respectively. For Foxp3 staining, PE-labelled anti-Mouse/Rat Foxp3 antibody (eBioscience, 12-5773-82, 1:100) was used. Guava easyCyte 8HT System and GuavaSoft software were used for the analysis.

### CD4^+^ T-cell separation and differentiation

Naive CD4^+^ T cells were isolated from the spleens of female C57BL/6 mice (8–9 weeks) by magnetic cell separation (Invitrogen). The cells were cultured in complete RPMI 1640 containing 10% FBS, L-glutamine (2 mM) and 2-mercaptoethanol (50 μM). The cells were activated with anti-CD3 (BioLegend, 100331, 2 μg ml^−1^) and anti-CD28 (BioLegend, 102112, 2 μg ml^−1^). Th1 cells were generated by the addition of IL-12 (10 ng ml^−1^) and anti-IL-4 (BioLegend, 504115, 10 μg ml^−1^). For Th17 differentiation, the cells received anti-IL-4 (10 μg ml^−1^) and anti-IFN-γ (BD Bioscience, 551216, 10 μg ml^−1^) plus a Th17 mixture containing TGF-β1 (3 ng ml^−1^), IL-6 (30 ng ml^−1^), TNF-α (10 ng ml^−1^) and IL-1β (10 ng ml^−1^). Polarization of Treg was induced by the addition of TGF-β1 (5 ng ml^−1^), IL-2 (10 ng ml^−1^) and anti-IFN-γ (10 μg ml^−1^).

### Reverse transcription and PCR

Total RNA was extracted with TRIzol (Invitrogen). The RNA was subjected to reverse transcription with random hexamer primer and Moloney murine leukemia virus reverse transcriptase (Promega). Real-time PCR was conducted in a LightCycler quantitative PCR apparatus (Stratagene) using the FastStart Universal SYBR Green Master (Rox). All the gene expression results are expressed as arbitrary units relative to expression of the gene encoding β-actin. The sequences of the primer pairs used are showed in Supplementary Table 1.

To detect the opioid receptors in OPCs and OLs, PCR was performed using diluted reverse transcription products, 35 cycles were used for DOR, KOR and MOR transcripts amplification, 27 cycles were used for PDGFRα, MBP and GAPDH transcripts amplification. All the products of RT-PCR were analysed by 1.5% agarose gel electrophoresis. The sequences of the primer pairs are showed in Supplementary Table 1.

### Isolation of primary astrocyte and microglia

Whole brains of newborn mice (P0–P2) were harvested and dissociated with Neural Tissue Dissociation Kit (Miltenyi). For astrocyte isolation, the whole-brain cells were suspended in complete DMEM containing 10% FBS and seeded onto 100-mm dishes. After 7 days (with medium changes at 24 h and then every 3 days), the cultures were trypsinized and re-plated. The cells from the cultures that had been passaged twice were used as astrocytes and purity was >95% as determined by immunostaining with anti-GFAP antibodies (Millipore, MAB360, 1:200). For microglia isolation, the whole-brain cells were sorted by anti-CD11b microbeads (Miltenyi) and purity was >95%. The cells were suspended in DMEM plus 10% FBS and seeded on 12-well plates. After 10 days of cultivation (with medium changes every 3 days), the cells were ready for LPS stimulation.

### OPC differentiation and immunocytochemistry

Neural progenitor cells (NPC) were isolated from the cerebral cortex of embryonic day 15.5 mouse embryos. NPCs were expanded as neurospheres in the DMEM/F12 medium containing 20 ng ml^−1^ EGF, 20 ng ml^−1^ bFGF and 2% B27. To generate OPCs, neurospheres were dissociated into single cells with accutase and plated on poly-ornithine plus laminin-coated plates in DMEM/F12 medium containing 10 ng ml^−1^ bFGF, 10 ng ml^−1^ PDGF-AA and 2% B27 for 2 days. To induce OPC differentiation, the cells were cultured in basal medium (DMEM/F12 containing 2% B27) with or without the KOR agonist U50488. After 5 days' differentiation, the cells were fixed and stained with anti-MBP antibody and secondary antibody conjugated to Alexa Fluor 488. Hoechst 33342 was used to identify cell nuclei. For cell imaging, we captured five pictures per well and detected the nuclei and MBP-positive cells using Operetta high content analysis system.

### Electron microscopy

The spinal cords isolated from paraformaldehyde (4%, w-v) perfused mice were fixed in PBS containing 2.5% glutaraldehyde for 2 h. Then the tissues were washed, fixed in 1% osmium tetroxide, dehydrated in acetone and embedded in EPON. Then 70-nm thin sections were cut with a diamond knife and mounted on copper slot grids coated with Formvar and stained with uranyl acetate and lead citrate for examination on JEM-1230 transmission electron microscope. The images were analysed in ImagePro for g-ratio measurements, approximately 200 axon and axon plus myelin units were measured for each group.

### OPC-DRG neuron co-culture

DRG neurons were prepared as previously reported^29^. In brief, DRGs were isolated from postnatal (P5–P10) mouse and incubated in papain (3 U ml^−1^, Sigma) and L-cysteine (0.36 mg ml^−1^) solution for 10 min at 37 °C. After careful removal of papain solution, DRGs were further incubated in collagenase II (100 U ml^−1^, Roche) and dispase II (2 U ml^−1^, Roche) solution for 10 min at 37 °C. After thorough washing, the dissociated DRG neurons were seeded at a density of 20,000 cells per well onto poly-D-lysine and laminin-coated 48-well dish and maintained in OL-medium (DMEM with B27, Glutamax, insulin (5 μg ml^−1^), transferrin (50 μg ml^−1^), 0.5% FBS, progesterone (0.2 μM), 3,3′,5-triiodo-L-thyronine (0.6 μM), putrescine (100 μM) and BSA (0.1 mg ml^−1^)), and fluorodeoxyuridine (10 μM) was added to remove contaminating glia cells. After 9 days, 3 × 10^4^ OPCs isolated from 0 to 2 postnatal day mouse cortices with anti-AN2 microBeads (Miltenyi) were added per well to DRGs, and co-cultures were maintained for another 9 days in OL-medium. In myelination experiments, drugs were added after the addition of OPCs. The cultures were fixed and stained with anti-NF-200 (Sigma, N4142, 1:1,000) and anti-MBP (Covance, SMI-94R, 1:500) antibodies. For cell imaging, we captured 10 pictures per well using Operetta high content analysis system and myelination was identified as neuritis double positive for MBP and NF-200 staining.

### Statistical analysis

The data are presented as means±s.e.m. Two-way analysis of variance test was used to assess the significance between treatment groups of EAE animals. For any given date, the EAE clinical scores were analysed using a non-parametric Mann–Whitney *U*-test. For other analyses, including gene expression, cell percentage and histological analysis, the significance was assessed by two-tailed Student's *t*-test. The *P* values <0.05 were considered statistically significant.

## Additional information

**How to cite this article:** Du, C. *et al*. Kappa opioid receptor activation alleviates experimental autoimmune encephalomyelitis and promotes oligodendrocyte-mediated remyelination. *Nat. Commun.* 7:11120 doi: 10.1038/ncomms11120 (2016).

## Supplementary Material

Supplementary Information Supplementary Figures 1-8 and Supplementary Table 1
