# Supplementary material for: Kappa opioid receptor activation alleviates experimental autoimmune encephalomyelitis and promotes oligodendrocyte-mediated remyelination
Source: Nat Commun. 2016 Apr 4;7:11120. doi: 10.1038/ncomms11120 (PMC4822006; doi:10.1038/ncomms11120)
Supplement: Supplementary Information — Supplementary Figures 1-8 and Supplementary Table 1 [file ncomms11120-s1.pdf]

Supplementary Figure 1. Expression of endogenous opioids and opioid receptors during the pathogenesis of EAE

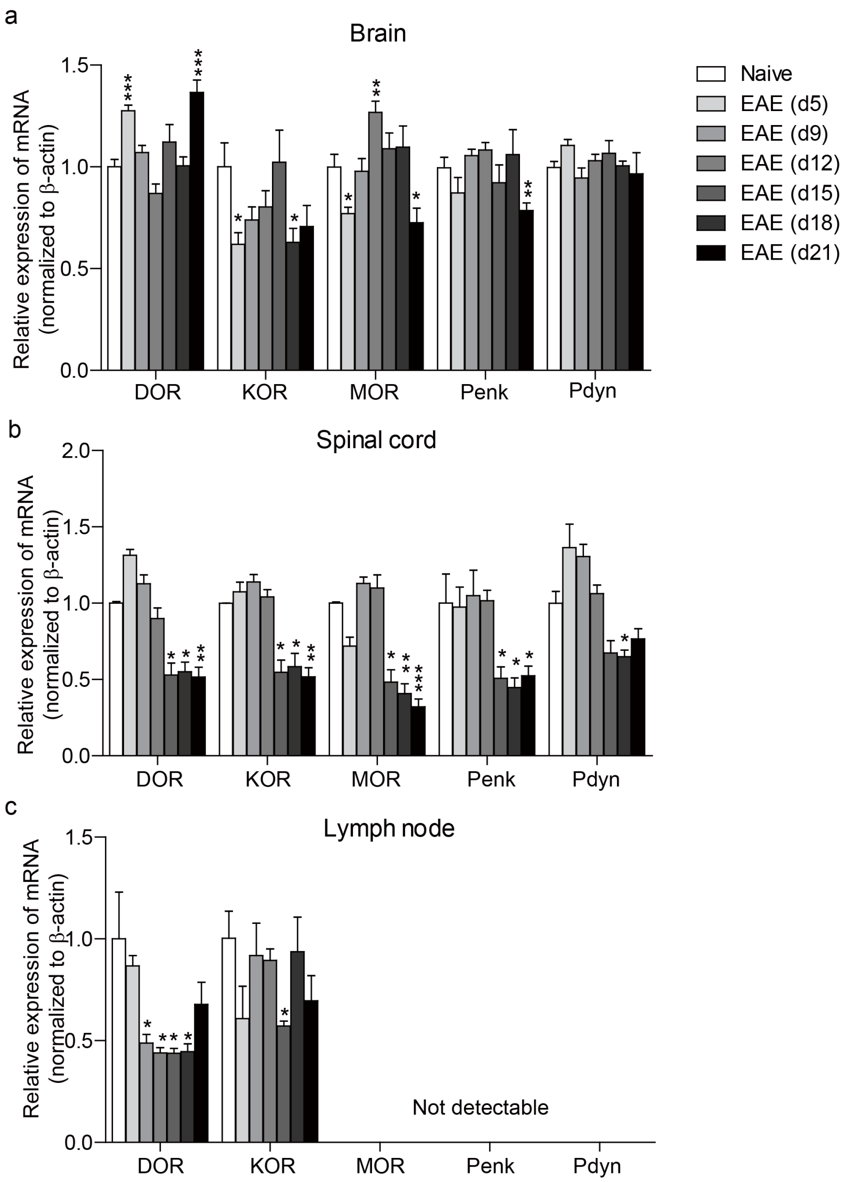

(**a**-**c**) Realtime q-PCR analysis of the expression changes of opioid receptors and their ligands in brain (**a**), spinal cord (**b**), and lymph node (**c**) of WT mice during EAE pathogenesis (Day 5-21 postimmunization). Results were normalized to β-actin expression in the same sample and then normalized to the naive control animals. Data are presented as means ± SEM (n≥5), *p<0.05, **p<0.01, and ***p<0.001 versus naive control (Student t test).

Supplementary Figure 2. Asimadoline alleviates clinical symptoms of EAE

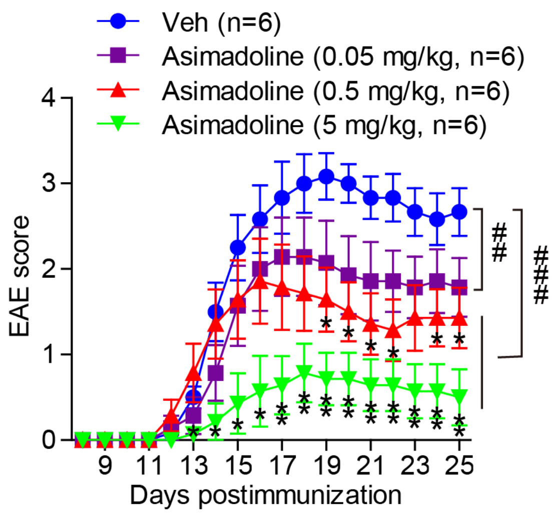

Clinical scores of WT-EAE mice treated with KOR agonist asimadoline (0.05, 0.5, or 5 mg kg^{-1}) or vehicle (saline) once daily via *i.p.* injection from day 3 postimmunization till the end of the study. Data are means ± SEM. ##$p<0.01$, ###$p<0.001$ (two-way ANOVA test), *$p<0.05$, **$p<0.01$ versus vehicle control (Mann-Whitney U test).

Supplementary Figure 3. U50488 treatment reduces leukocyte infiltration and demyelination in EAE animals

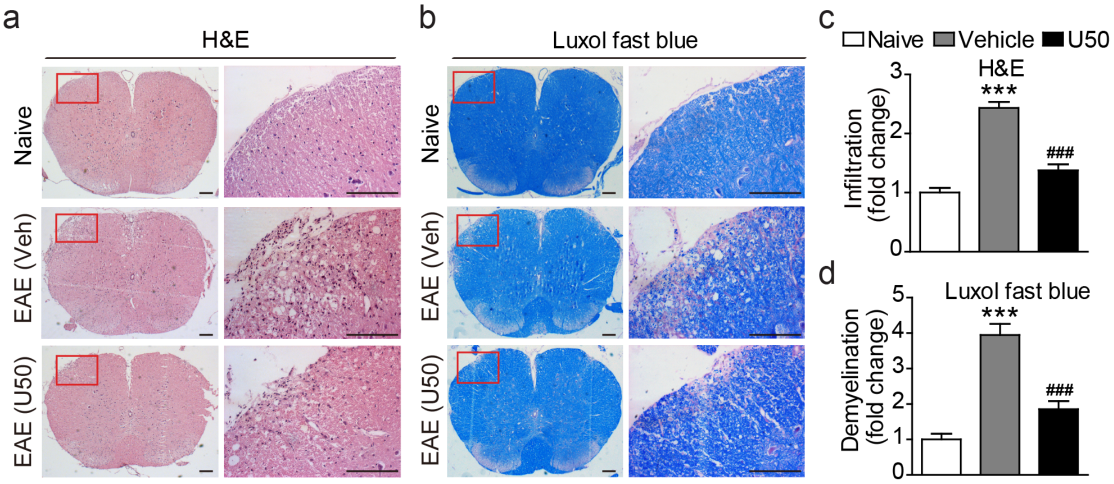

(**a**) H&E and (**b**) Luxol fast blue staining of paraffin sections of spinal cords isolated from naive, vehicle-, or U50488 (U50, 1.6 mg kg^{-1})-treated WT-EAE mice on day 17. Scale bars: 200 μm. (**c**,**d**) Quantification of CNS infiltrates (**c**) and the amount of demyelination (**d**) presented in (**a**) and (**b**). Data are means ± SEM. Three mice from each group were sacrificed, and 15 sections from each mouse were analyzed. $^{***}p<0.001$ versus naive, $^{\#\#\#}p<0.001$ versus vehicle-EAE (Student *t* test).

Supplementary Figure 4. U50488 treatment or KOR deficiency does not affect the percentage of the major subgroups of immune cells

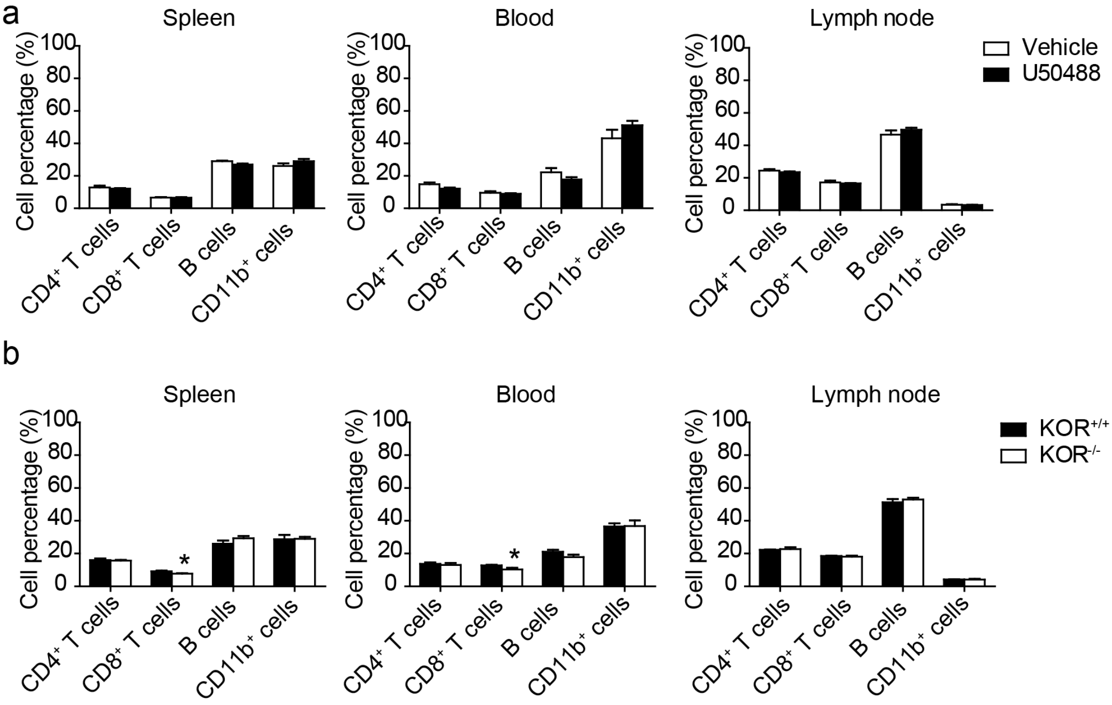

(**a**) Leukocytes in the spleen, blood, and lymph nodes were isolated from WT-EAE mice treated with vehicle or U50488 (1.6 mg kg^{-1}) at day12 postimmunization and stained for $CD4^+$ T cells, $CD8^+$ T cells, B cells (B220) and $CD11b^+$ cells. Cell percentages were determined with flow cytometry analysis. Data are means ± SEM (n=5). (**b**) Leukocytes in the spleen, blood, and lymph nodes were isolated from WT-EAE or $KOR^{-/-}$-EAE mice at day 12 postimmunization and stained for $CD4^+$ T cells, $CD8^+$ T cells, B cells and $CD11b^+$ cells. Cell percentages were determined with flow cytometry analysis. Data are means ± SEM (n=5), *$p<0.05$ versus WT control (Student *t* test).

Supplementary Figure 5. KOR activation or knockout does not affect Th1 and Th17 development *in vivo*

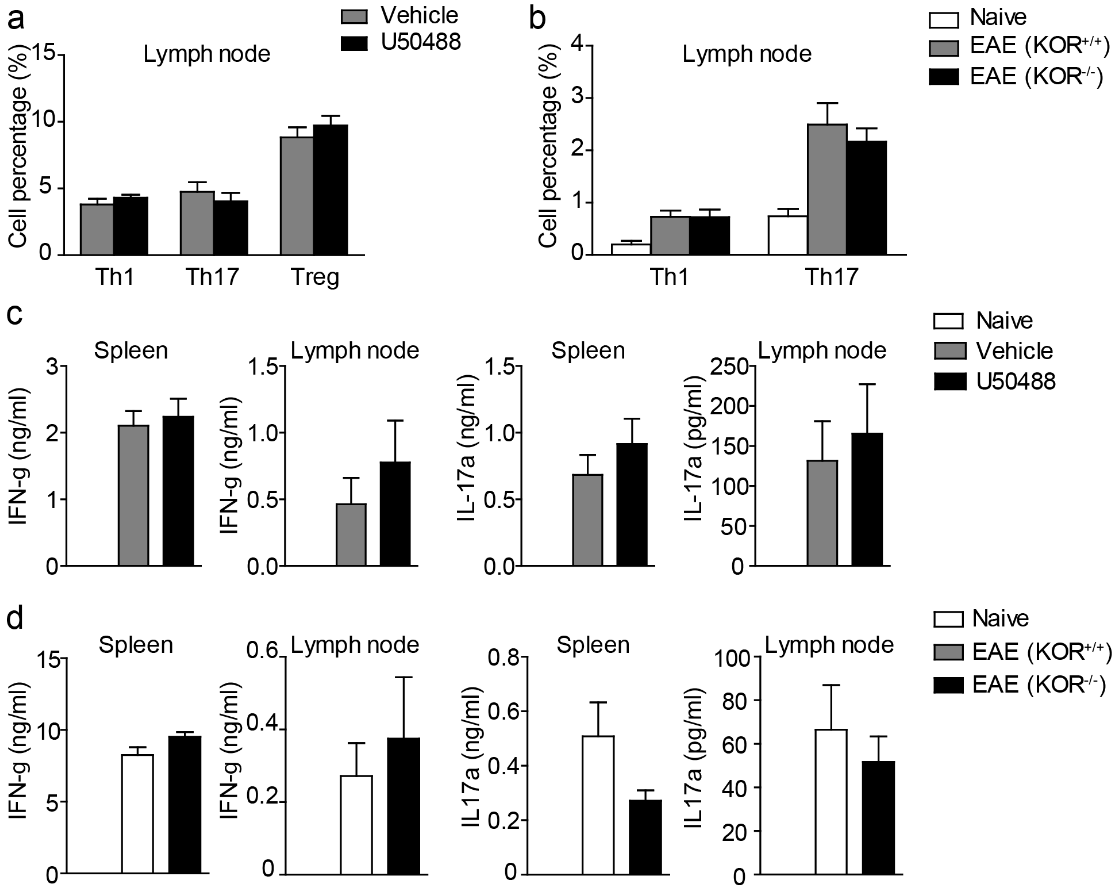

(**a**,**c**) Leukocytes were isolated from lymph node and spleen of WT-EAE mice treated with U50488 (1.6 mg kg^{-1}) or vehicle on day 12 postimmunization. Th1, Th17, and Treg cells were analyzed with FACS by intracellular staining of IFN-γ, IL-17A, and Foxp3 respectively, in the $CD4^+$ gate (**a**). Cytokines (IFN-γ and IL-17A) in the supernatant after MOG restimulation were detected by ELISA (**c**). Data are means ± SEM (n≥7). (**b**,**d**) Leukocytes were isolated from lymph node and spleen of WT- or $KOR^{-/-}$-EAE mice on day 12 postimmunization. (**b**) Th1 and Th17 cells were analyzed with FACS by intracellular staining of IFN-γ and IL-17A, respectively, in the $CD4^+$ gate. (**d**) IFN-γ and IL-17A in the supernatant after MOG restimulation were detected by ELISA. Data are means ± SEM (n≥5).

Supplementary Figure 6. Effect of KOR signaling in cytokine activated mouse brain vascular endothelia cells *in vitro*

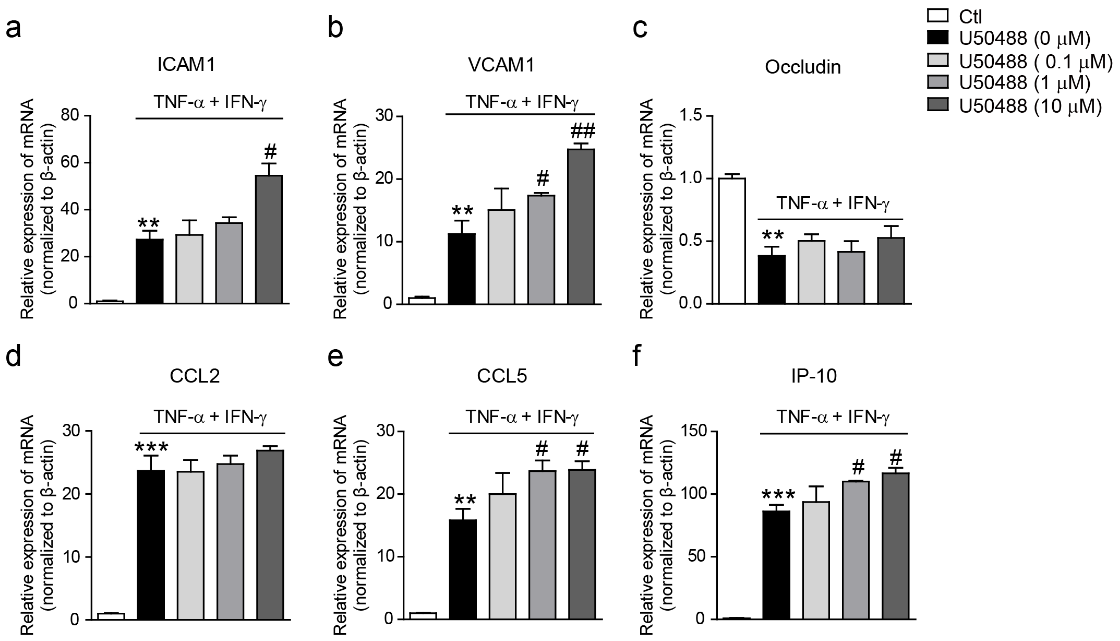

(**a**-**f**) Mouse brain microvascular endothelial bEnd.3 cells were treated with U50488 for 30 min and then stimulated with IFN-γ (50 ng ml^{-1}) plus TNF-α (50 ng ml^{-1}) for 6 h in the presence of U50488. Cells were harvested for RNA extraction. Expression of chemokines and cell adhesion molecus were analyzed with real-time PCR. Results were normalized to β-actin expression in the same sample and then normalized to the control group. Data are presented as means ± SEM. $^{**}p<0.01$, $^{***}p<0.001$ versus control, $^{\#}p<0.05$, $^{\#\#}p<0.01$ versus none U50488 treated group (Student *t* test).

Supplementary Figure 7. Effect of KOR signaling in astrocytes activation *in vitro*

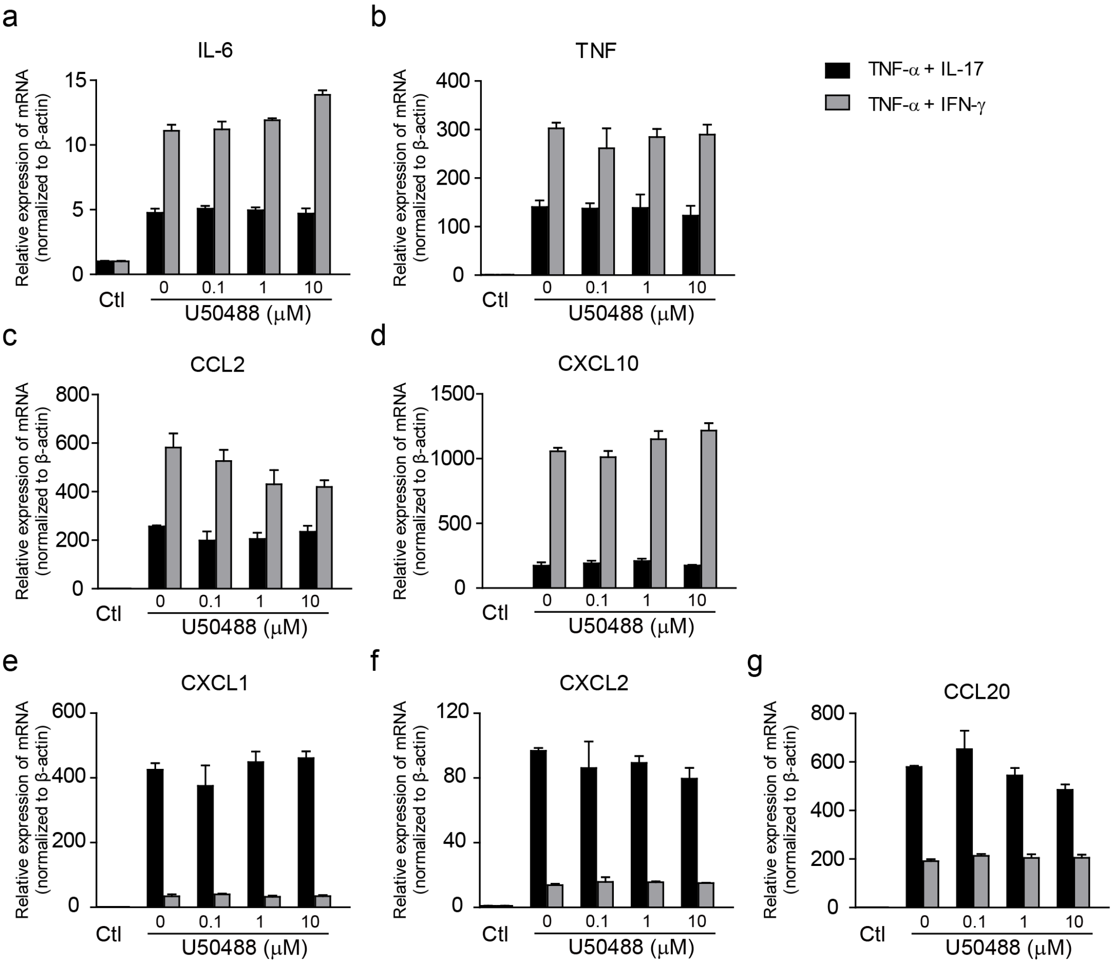

(**a**-**g**) Primary astrocytes were treated with U50488 for 30 min, and then IL-17A (50 ng ml^{-1}) plus TNF-α (10 ng ml^{-1}) or IFN-γ (10 ng ml^{-1}) plus TNF-α (10 ng ml^{-1}) were added into the cultures and incubated for 6 h. Cells were then harvested for RNA extraction. Expression of proinflammatory cytokines and chemokines were analyzed with real-time PCR. Results were normalized to β-actin expression in the same sample and then normalized to the control group. Data are presented as means ± SEM.

Supplementary Figure 8. Effect of KOR signaling in microglia activation *in vitro*

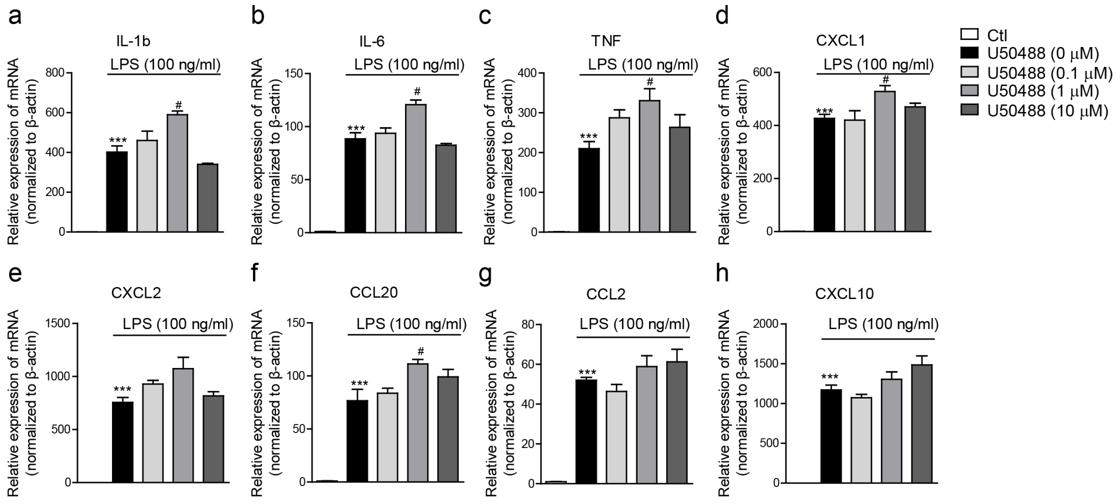

(**a**-**h**) Primary microglia were treated with U50488 for 30 min and then stimulated with 100 ng ml^{-1} LPS for 2 h. Cell were harvested for RNA extraction. Expression of proinflammatory cytokines and chemokines were analyzed with real-time PCR. Results were normalized to β-actin expression in the same sample and then normalized to the control group. Data are presented as means ± SEM $^{***}p<0.001$ versus control, $^{\#}p<0.05$, versus none U50488 treated group (Student *t* test).

Supplementary Table 1. PCR Primers

Gene	Forward primer	Reverse primer
β-actin	GGCTGTATTCCCCTCCATCG	CCAGTTGGTAACAATGCCATGTT
DOR	GCTGGTGGACATCAATCGG	GCGTAGAGAACCGGGTTGA
KOR	TCCCCAACTGGGCAGAATC	GACAGCGGTGATGATAACAGG
MOR	TGGCTCCTGGCTCAACTTG	CAGCGTGCTAGTGGCTAAGG
Penk	CCCAGGCGACATCAAT	GCAAGTGGCTCTCATCCT
Pdyn	CAGTGAGGATTCAGGATGGG	CGTCAGGGTGAGAAAAGATGA
PDGFRa	TCCATGCTAGACTCAGAAGTCA	TCCCGG- TGGACACAATTTTTC
MBP	TGACACCTCGAACACCACCTC	CCTTGAATCCCTTGTGAGCC
GAPDH	AGGTCGGTGTGAACGGATTTG	TGTAGACCATGTAGTTGAGGTCA
TNF	CTCAAAATTCGAGTGACAAGCCT	GGTTGTCTTTGAGATCCATGCC
IL-6	ACCACGGCCTTCCCTACTTC	GAATTGCCATTGCACAACTCTT
IL-1b	AGGCAGGCAGTATCACTCATTG	GTCACACACCAGCAGGTTATCA
CXCL1	CTTGCCTTGACCCTGAAGCTC	AGCAGTCTGTCTTCTTTCTCCGT
CXCL2	CCCCCTGGTTCAGAAAATCA	GCTCCTCCTTTCCAGGTCAGT
CXCL10	GCTGCAACTGCATCCATATCG	TGCTCATCATTCTTTTTCATCGT
CCL2	GCTGACCCCAAGAAGGAATG	AGGTGGTTGTGGAAAAGGTAGTG
CCL20	CAAGCGTCTGCTCTTCCTTG	TGGATCAGCGCACACAGATT
CCL5	CCCTCACCATCATCCTCACT	GGGAAGCGTATACAGGGTCA
VCAM1	TGTTTGGCTCCAGACATTTACC	GGAGGGATGTACAGAGATCGTTG
ICAM1	GGAGGGATGTACAGAGATCGTTG	GGGGTGTCGAGCTTTGGGA
Occludin	GTATAAGAGCTTACAGGCAGAACTAGA	TCTGTCATAATCTCCCACCATCC
